# CLIMB (the Cloud Infrastructure for Microbial Bioinformatics): an online resource for the medical microbiology community

**DOI:** 10.1099/mgen.0.000086

**Published:** 2016-09-20

**Authors:** Thomas R. Connor, Nicholas J. Loman, Simon Thompson, Andy Smith, Joel Southgate, Radoslaw Poplawski, Matthew J. Bull, Emily Richardson, Matthew Ismail, Simon Elwood- Thompson, Christine Kitchen, Martyn Guest, Marius Bakke, Samuel K. Sheppard, Mark J. Pallen

**Affiliations:** ^1^​ Cardiff University School of Biosciences, The Sir Martin Evans Building, Cardiff University, Cardiff CF10 2AX, UK; ^2^​ Institute of Microbiology and Infection, University of Birmingham, Birmingham B15 2TT, UK; ^3^​ IT Services (Research Computing), University of Birmingham, Birmingham B15 2TT, UK; ^4^​ Centre for Scientific Computing, University of Warwick, Coventry CV4 7AL, UK; ^5^​ College of Medicine, Swansea University, Swansea, UK; ^6^​ Advanced Research Computing@Cardiff (ARCCA), Cardiff University, UK; ^7^​ Microbiology and Infection Unit, Warwick Medical School, University of Warwick, Coventry, UK; ^8^​ The Milner Centre for Evolution, Department of Biology and Biochemistry, University of Bath, Bath BA2 7AY, UK

**Keywords:** cloud computing, bioinformatics, infrastructure, metagenomics, population genomics, virtual laboratory

## Abstract

The increasing availability and decreasing cost of high-throughput sequencing has transformed academic medical microbiology, delivering an explosion in available genomes while also driving advances in bioinformatics. However, many microbiologists are unable to exploit the resulting large genomics datasets because they do not have access to relevant computational resources and to an appropriate bioinformatics infrastructure. Here, we present the Cloud Infrastructure for Microbial Bioinformatics (CLIMB) facility, a shared computing infrastructure that has been designed from the ground up to provide an environment where microbiologists can share and reuse methods and data.

## Data Summary

The paper describes a new, freely available public resource and therefore no data have been generated. The resource can be accessed at http://www.climb.ac.uk. Source code for software developed for the project can be found at http://github.com/MRC-climb/.

## Impact Statement

Technological advances mean that genome sequencing is now relatively simple, quick and affordable. However, handling large genome datasets remains a significant challenge for many microbiologists, with substantial requirements for computational resources and expertise in data storage and analysis. This has led to fragmentary approaches to software development and data sharing that reduce the reproducibility of research and limits opportunities for bioinformatics training. Here, we describe a nationwide electronic infrastructure that has been designed to support the UK microbiology community, providing simple mechanisms for accessing large, shared, computational resources designed to meet the bioinformatics needs of microbiologists.

## Introduction

Genome sequencing has transformed the scale of questions that can be addressed by biological researchers. Since the publication of the first bacterial genome sequence over 20 years ago (Fleischmann *et al.,* 1995), there has been an explosion in the production of microbial genome sequence data, fuelled most recently by high-throughput sequencing (Loman & Pallen, 2015). This has placed microbiology at the forefront of data-driven science (Marx, 2013). As a consequence, there is now huge demand for physical and computational infrastructures to produce, analyse and share microbiological software and datasets and a requirement for trained bioinformaticians who can use genome data to address important questions in microbiology (Chang, 2015). It is worth stressing that microbial genomics, with its focus on the extensive variation seen in microbial genomes, brings challenges altogether different from the analysis of the larger but less variable genomes of humans, animals or plants.

One solution to the data-deluge challenge is for every microbiology research group to establish their own dedicated bioinformatics hardware and software. However, this entails considerable upfront infrastructure costs and great inefficiencies of effort, while also encouraging a working-in-silos mentality, which makes it difficult to share data and pipelines and thus hard to replicate research. Cloud computing provides an alternative approach that facilitates the use of large genome datasets in biological research (Chang, 2015).

The cloud-computing approach incorporates a shared online computational infrastructure, which spares the end user from worrying about technical issues such as the installation, maintenance and, even, the location of physical computing resources, together with other potentially troubling issues such as systems administration, data sharing, scalability, security and backup. At the heart of cloud computing lies *virtualization*, an approach in which a physical computing set-up is re-purposed into a scalable system of multiple independent *virtual machines*, each of which can be pre-loaded with software, customized by end users and saved as *snapshots* for re-use by others on the infrastructure. Ideally, such an infrastructure also provides large-scale data storage and compute capacity on demand, reducing costs to the public purse by optimizing utilization of hardware and avoiding resources sitting idle while still capitalizing on the economies of scale.

The potential for cloud computing in biological research has been recognized by funding organizations and has seen the development of nationwide resources such as iPlant (Goff *et al.,* 2011) (now CyVerse), NECTAR (http://nectar.org.au) and XSEDE (Towns *et al.,* 2014) that provide researchers with access to large cloud infrastructures. Here, we describe a new facility, designed specifically for microbiologists, to provide a computational and bioinformatics infrastructure for the UK’s academic medical microbiology community, facilitating training, access to hardware and sharing of data and software.

## Resource overview

**Fig. 1.**
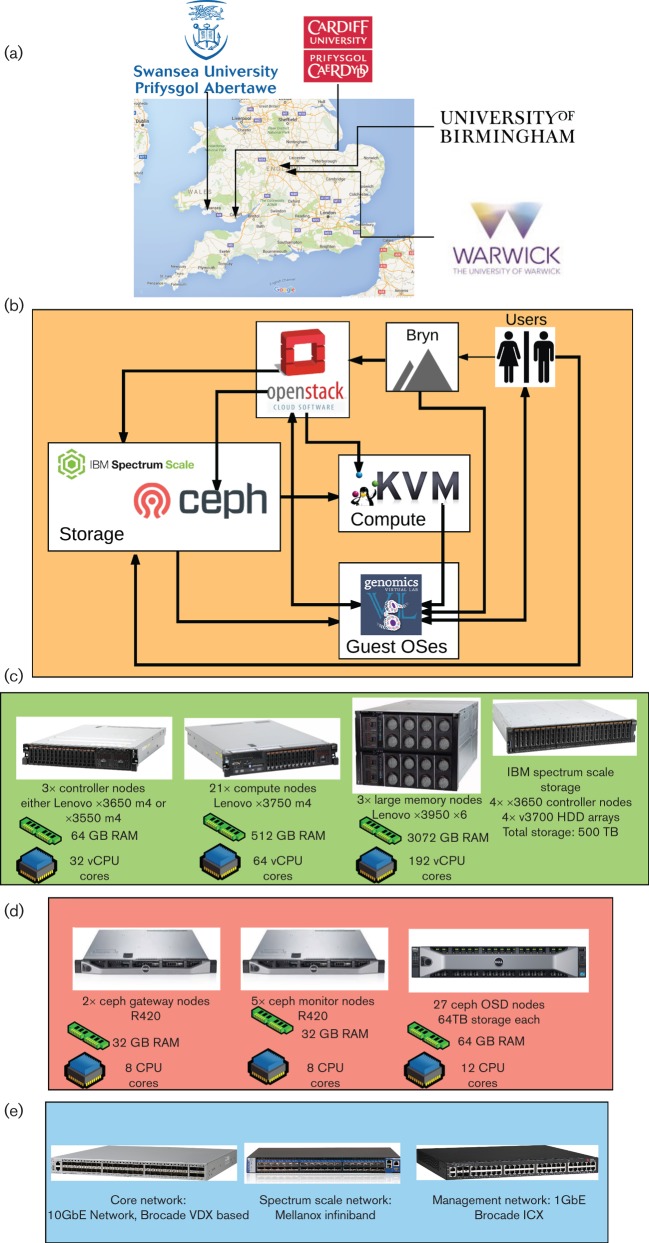
Overview of the system. (a) The sites where the computational hardware is based. (b) High-level overview of the system and how the different software components connect to one another. (c) Compute hardware present at each of the four sites. (d) Hardware comprising the Ceph storage system at each site. (e) Type and role of network hardware used at each site.

The Cloud Infrastructure for Microbial Bioinformatics (CLIMB) facility is intended as a general solution to pressing issues in big-data microbiology. The resource comprises a core physical infrastructure (Fig. 1), combined with three key features making the cloud suitable for microbiologists.

First, CLIMB provides a single environment, complete with pipelines and datasets that are co-located with computing resource. This makes the process of accessing published packages and sequence data simpler and faster, improving reuse of software and data.

Second, CLIMB has been designed with training in mind. Rather than having trainees configure personal laptops or face challenges in gaining access to shared high-performance computing resources, we provide training images on virtual machines that have all the necessary software installed and we provide each trainee with his or her own personal server to continue learning after the workshop concludes.

Third, by bringing together expert bioinformaticians, educators and biologists in a unified system, CLIMB provides an environment where researchers across institutions can share data and code, permitting complex projects iteratively to be remixed, reproduced, updated and improved.

The CLIMB core infrastructure is a cloud system running the free open-source cloud operating system OpenStack (http://www.openstack.org). This system allows us to run over 1000 virtual machines at any one time, each preconfigured with a standard user configuration. Across the cloud, we have access to over 78 terabytes of RAM. Specialist users can request access to one of our 12 high-memory virtual machines each with 3 terabytes of RAM for especially large, complex analyses (Fig. 1). The system is spread over four sites to enhance its resilience and is supported by local scratch storage of 500 terabytes per site employing IBM’s Spectrum Scale storage (formerly GPFS). The system is underpinned by a large shared object storage system that provides approximately 2.5 petabytes of data storage, which may be replicated between sites. This storage system, running the free open-source Ceph system (http://www.redhat.com/en/technologies/storage/ceph), provides a place to store and share very large microbial datasets – for comparison, the bacterial component of the European Nucleotide Archive is currently around 400 terabytes in size. The CLIMB system can be coupled to sequencing services; for example, sequence data generated by the MicrobesNG service (http://www.microbesng.uk) have been made available within the CLIMB system.

## Resource performance

**Fig. 2.**
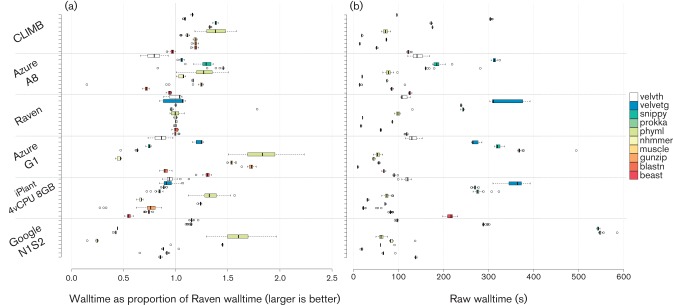
Relative performance of virtual machines running on cloud services, compared to the Cardiff University HPC system, Raven. (a) Values for each package are the mean of the wall time taken for 10 runs performed on Raven, divided by the mean wall time of 40 runs undertaken on the virtual machine on the named service. Values greater than 1 are faster than Raven, values less than 1 are slower. (b) The raw wall time values for the named software on each of the systems. The data generated as part of the benchmarking exercise is included in Supplementary File 1.

To assess the performance of the CLIMB system in comparison to traditional high-performance computing (HPC) systems and similar cloud systems, we undertook a small-scale benchmarking exercise (Fig. 2). Compared to the Raven HPC resource at Cardiff (running Intel processors a generation behind those in CLIMB), performance on CLIMB was generally good, offering a relative increase in performance of up to 38 % on tasks commonly undertaken by microbial bioinformaticians. The CLIMB system also compares well to cloud servers from major providers, offering better aggregate performance than Microsoft Azure A8 and Google N1S2 virtual machines. The results also reveal a number of features that may be relevant to where a user chooses to run their analysis. CLIMB performs worse than Raven when running beast (Drummond & Rambaut, 2007), and provides a limited increase in performance for the package nhmmer, suggesting that while it is possible to run these analyses on CLIMB, other resources – such as local HPC facilities – might be more appropriate as these are optimized for computationally intensive workloads. Conversely, the largest performance increases are observed for Prokka (Seemann, 2014), Snippy and PhyML (Guindon *et al.,* 2010), which encompass some of the most commonly used analyses undertaken in microbial genomics. It is also interesting to note that both commercial clouds offer excellent performance relative to Raven for two workloads: muscle (Edgar, 2004) and PhyML. The source of this performance is difficult to predict, but it is possible that these workloads may be more similar to the sort of workloads that these cloud services have been designed to handle. On the basis of the performance results more generally, however, CLIMB is likely to offer a number of performance benefits over local resources for many microbial bioinformatics workloads.

## Providing a single environment for training, data and software sharing

**Fig. 3.**
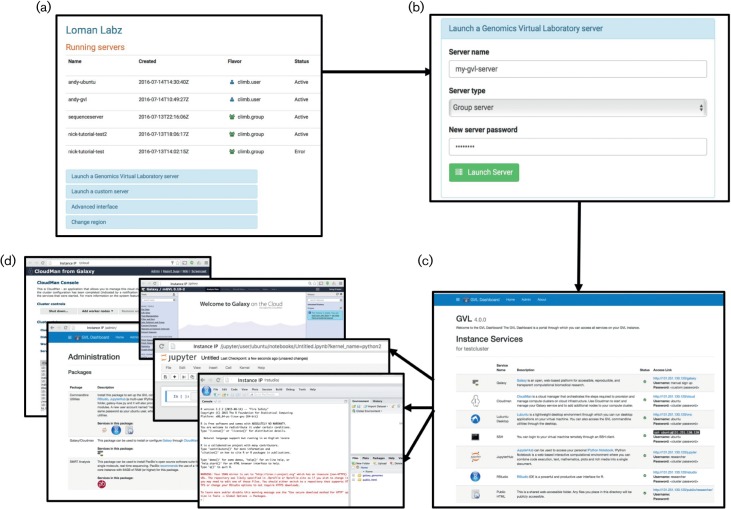
CLIMB virtual machine launch workflow. A user, on logging in to the Bryn launcher interface, is presented with a list of the virtual machines they are running and are able to stop, reboot or terminate them (a). Users launch a new Genomics Virtual Laboratory (GVL) server with a minimal interface, specifying a name, the server ‘flavour’ (user or group) and an access password (b). On booting, the user accesses a webserver running on the GVL instance, which gives access to various services that are started automatically (c). The GVL provides access to a Cloudman, a Galaxy server, an administration interface, Jupyter notebook and RStudio (d, top to bottom).

The CLIMB system is accessed through the Internet, via a simple set of web interfaces enabling the sharing of software on virtual machines (Fig. 3). Users request a virtual machine via a web form. Each virtual machine makes available the microbial version of the Genomics Virtual Laboratory (Fig. 3) (Afgan *et al.,* 2015). This includes a set of web tools [Galaxy (Pond *et al.,* 2009), Jupyter Notebook (Perez, 2015) and RStudio, with an optional PacBio SMRT portal], as well as a set of pipelines and tools that can be accessed via the command line. This standardized environment provides a common platform for teaching, while the base image provides a versatile platform that can be customized to meet the needs of individual researchers. To provide user support and documentation a CLIMB discussion forum (http://discourse.climb.ac.uk) is available. The forum contains a number of tutorials demonstrating functionality. Initial tutorials cover topics including shotgun metagenomics, genome-wide association studies, ancient DNA, the Nullarbor public health microbiology pipeline and setting up a simple blast server with SequenceServer (Priyam *et al.,* 2015).

## System access

Users register at our website, using their UK academic credentials (http://bryn.climb.ac.uk/). Upon registration, users have one of two modes of access: the first is to launch an instance running a preconfigured virtual machine, with a set of predefined pipelines or tools, which includes the Genomics Virtual Laboratory. The second option is aimed at expert bioinformaticians and developers who may want to be able to develop their own virtual machines from a base image – to enable this we also allow users to access the system via a dashboard, similar to that provided by Amazon Web Services, where users can specify the size and type of virtual machine that they would like, with the system then provisioning this up on demand. To share the resource fairly, users will have individual quotas that can be increased on request. Irrespective of quota size, access to the system is free of charge to UK academic users.

## Future directions and wider impact

CLIMB is likely to accumulate a library of images and datasets that will be of wide use to researchers within the UK and elsewhere. Currently it does not provide a simple system to export instances or data. However, it is already possible to export images and data from CLIMB, and we plan to develop systems to enable the rapid, simple sharing of virtual machines and data with other clouds (such as Amazon Web Services). While the computational resources are principally for UK researchers (and international collaborators working with UK-based Principal Investigators), by making virtual machines and data available to other services we provide a key mechanism for the international community to benefit from the resource. Nor is CLIMB functioning in a vacuum; the project is already engaged with academic cloud providers elsewhere in the world (such as NECTAR), and we are actively working with the European Bioinformatics Institute to examine ways in which virtual machines and complete datasets can be better shared and reused – another key output that is likely to be of considerable value to the international community. One of the challenges in this area is the fact that virtual machine images are monolithic entities that may package operating system software, installed packages, scripts and datasets into a single unit. However, recent advances in virtualization enable sharing of software and dependencies via lightweight ‘containers’ that are considerably smaller than virtual machines. Platforms such as Docker (https://www.docker.com/) and rkt (https://coreos.com/rkt/) provide new approaches that may be suitable for sharing complex bioinformatics pipelines without the need to package the operating system software. Such approaches may make it easier to integrate pipelines from multiple sources on a single virtual machine. As part of the development of CLIMB we hope to be able to support containers soon. Finally, CLIMB has been designed to allow the addition of hardware and other sites. It is our hope that as the system is used, it will also be built upon – expanding both its computational capacity and the number of sites involved – to extend the community that it is able to serve, adding in international sites and capacity for supporting researchers examining questions in other areas of data-intensive biology.

## Conclusion

CLIMB is probably the largest computer system dedicated to microbiology in the world. The system has already been used to address microbiological questions featuring bacteria (Connor *et al*., 2015) and viruses (Quick *et al.,* 2016). CLIMB has been designed from the ground up to meet the needs of microbiologists, providing a core infrastructure that is presented in a simple, intuitive way. Individual elements of the system – such as the large shared storage and extremely large memory systems – provide capabilities that are usually not available locally to microbiologists within most UK institutions, while the shared nature of the system provides new opportunities for data and software sharing that have the potential to enhance research reproducibility in data-intensive biology. Cloud computing clearly has the potential to revolutionize how biological data are shared and analysed. We hope that the microbiology research community will capitalize on these new opportunities by exploiting the CLIMB facility.

## Supplementary Data

97 Supplementary File 1
